# Inhibition of astroglial NF-kappaB enhances oligodendrogenesis following spinal cord injury

**DOI:** 10.1186/1742-2094-10-92

**Published:** 2013-07-23

**Authors:** Valerie Bracchi-Ricard, Kate L Lambertsen, Jerome Ricard, Lubov Nathanson, Shaffiat Karmally, Joshua Johnstone, Ditte G Ellman, Beata Frydel, Dana M McTigue, John R Bethea

**Affiliations:** 1 The Miami Project to Cure Paralysis, University of Miami, Miami FL 33136, USA; 2 Department of Neurobiology Research, Institute of Molecular Medicine, University of Southern Denmark, 5000, Odense, C, Denmark; 3 Department of Molecular and Cellular Medicine, Miller School of Medicine, University of Miami, Miami FL 33136, USA; 4 Department of Neuroscience, The Center for Brain and Spinal Cord Repair, The Ohio State University, 795 12th Avenue, Columbus OH 43210, USA

**Keywords:** NF-kappaB, Spinal cord injury, Astrocyte, Oligodendrocyte, Microglia, CXCR4, TNFR2, Toll-like receptor

## Abstract

**Background:**

Astrocytes are taking the center stage in neurotrauma and neurological diseases as they appear to play a dominant role in the inflammatory processes associated with these conditions. Previously, we reported that inhibiting NF-κB activation in astrocytes, using a transgenic mouse model (GFAP-IκBα-dn mice), results in improved functional recovery, increased white matter preservation and axonal sparing following spinal cord injury (SCI). In the present study, we sought to determine whether this improvement, due to inhibiting NF-κB activation in astrocytes, could be the result of enhanced oligodendrogenesis in our transgenic mice.

**Methods:**

To assess oligodendrogenesis in GFAP-IκBα-dn compared to wild-type (WT) littermate mice following SCI, we used bromodeoxyuridine labeling along with cell-specific immuno-histochemistry, confocal microscopy and quantitative cell counts. To further gain insight into the underlying molecular mechanisms leading to increased white matter, we performed a microarray analysis in naïve and 3 days, 3 and 6 weeks following SCI in GFAP-IκBα-dn and WT littermate mice.

**Results:**

Inhibition of astroglial NF-κB in GFAP-IκBα-dn mice resulted in enhanced oligodendrogenesis 6 weeks following SCI and was associated with increased levels of myelin proteolipid protein compared to spinal cord injured WT mice. The microarray data showed a large number of differentially expressed genes involved in inflammatory and immune response between WT and transgenic mice. We did not find any difference in the number of microglia/leukocytes infiltrating the spinal cord but did find differences in their level of expression of toll-like receptor 4. We also found increased expression of the chemokine receptor CXCR4 on oligodendrocyte progenitor cells and mature oligodendrocytes in the transgenic mice. Finally TNF receptor 2 levels were significantly higher in the transgenic mice compared to WT following injury.

**Conclusions:**

These studies suggest that one of the beneficial roles of blocking NF-κB in astrocytes is to promote oligodendrogenesis through alteration of the inflammatory environment.

## Background

Spinal cord injury (SCI) is a devastating condition affecting millions of people worldwide. Following the initial trauma to the spinal cord, with loss of cells at the site of impact, a second phase injury occurs characterized in part by secretion of cytokines and chemokines produced at the lesion site leading to recruitment of peripheral leukocytes to the injury [1]. While an inflammatory response is necessary to clear debris at the site of injury it, if uncontrolled, leads to an enlargement of the initial lesion, with additional axonal damage, oligodendrocyte cell death and demyelination with concomitant increased loss of neurological function. The loss of oligodendrocytes, however, may be replaced by proliferating nerve/glial antigen 2^+^ (NG2) cells, also known as oligodendrocyte precursor cells (OPCs) [2]. These OPCs are able to migrate to the injury site and differentiate into mature myelinating oligodendrocytes if the environment is permissive [3]. The lack of effective remyelination is often due to the presence of oligodendrocyte differentiation inhibitors in the injury environment, which can originate from astrocytes, demyelinated axons or myelin debris [4,5]. Until recently, the contribution of astrocytes to demyelinating diseases was underestimated. However, our laboratory and others have now established a prominent role of astrocytes in vivo in the pathogenesis of experimental autoimmune encephalomyelitis (EAE) [6-8] and axonal degeneration [9] and in vitro an increasing number of astroglial-derived factors have been identified that modulate myelination processes [7,10,11].

One of the ways astrocytes respond to injury is by producing cytokines and chemokines, many of which are regulated by NF-κB. To study the role of astroglial NF-κB in the pathogenesis of SCI, we previously generated transgenic mice (GFAP-IκBα-dn) in which NF-κβ is specifically inactivated in astrocytes by overexpression of a truncated form of the inhibitor IκBα (IκBα-dn) under the control of the glial fibrillary acidic protein (GFAP) promoter [12]. In this previous study, we demonstrated that blocking NF-κB activation in astrocytes resulted in reduced expression of cytokines and chemokines such as CXCL10, CCL2 and transforming growth factor beta, and in a smaller lesion volume and increased white matter sparing along with a significant improvement in locomotor function following SCI. Further studies showed that inhibition of astroglial NF-κB promoted axonal sparing and sprouting of supraspinal and propriospinal axons, which are essential for locomotion [13]. In a brain injury model astroglial NF-κB was also found to play a central role in directing immune-glial interactions by regulating the expression of CCL2 through STAT2 [9]. One explanation for the observed larger volume of white matter in our transgenic mice could be a reduction in oligodendrocyte cell death or an increase in oligodendrogenesis. Here, we are addressing the role of astroglial NF-κB in regulating oligodendrogenesis in the chronically injured spinal cord.

## Methods

### Mice

Adult (3 to 4 months) female GFAP-IκBα-dn (IκBα-dn) transgenic mice were generated and characterized in our laboratory [12]. All animals, IκBα-dn and wild-type (WT) littermates (LM), were kept as a colony in a virus/antigen-free environment at the University of Miami Miller School of Medicine, Miami, FL, USA. IκBα-dn mice were obtained by breeding heterozygous IκBα-dn males with WT females. Mice were housed under diurnal lightning conditions and allowed free access to food and water.

### Induction of spinal cord injury

Surgeries were performed at the Animal and Surgical Core Facility of the Miami Project to Cure Paralysis according to protocols approved by the Institutional Animal Care and Use Committee of the University of Miami. Contusion injury was induced with the Infinite Horizon Device (Precision Systems and Instrumentation LLC, Kentucky, USA). Female IκBα-dn (21.5 ± 2.7 g) and WT LM (21.0 ± 2.8 g) mice were anesthetized intraperitoneally (i.p.) using a ketamine (100 mg/kg, VEDCO Inc., Saint Joseph, MO, USA)/xylazine (10 mg/kg, VEDCO) cocktail, and a laminectomy was performed at the vertebral level T9. The contusion device was lowered onto the spinal cord at a predetermined impact force of 50 kdynes (moderate injury) and the mice were injured by a rapid displacement of the impounder resulting in a spinal cord displacement of 400 to 500 μm. Immediately after surgery, mice were sutured and injected subcutaneously (s.c.) with 1 ml lactated Ringer’s Injection USP (B. Braun, L7502, Bethlehem, PA, USA) to prevent dehydration and housed separately in a recovery room, where their post-surgical health status was observed. Thereafter, mice were returned to the conventional animal facility, where they were observed bi-daily for activity level and general physical condition. Manual bladder expression was performed twice a day until bladder function was regained. In addition, mice received s.c. prophylactic injections of antibiotic gentamicin (40 mg/kg, Hospira Inc., Lake Forest, IL, USA) for 7 days following SCI to prevent urinary tract infections. Mice were allowed 3 days, 3, 6 or 7 weeks survival.

### Bromodeoxyuridine injections and tissue processing

Mice in the 7 weeks survival group were injected i.p. with bromodeoxyuridine (BrdU; 50 μg/g body weight; Sigma, St. Louis, MO, USA) once a day for 7 days starting at week 5 post-SCI and were allowed to survive for 1 more week. Then the mice, naïve, 3 days, 6 and 7 weeks survival, were deeply anesthetized and perfused through the left ventricle using ice cold 0.01 M PBS followed by ice cold 4% paraformaldehyde (PFA) in PBS. The spinal cords were post-fixed in 4% PFA followed by immersion in 25% sucrose in PBS overnight. Spinal cords were cut into 1-cm segments centered on the injury site and then embedded in optimal cutting temperature (OCT) compound (VWR International, Arlington Heights, IL, USA), frozen and cut into 10 series of 25 μm transverse cryostat sections. Sections were stored at -20°C until further use.

### Immunohistochemistry

Antibodies used for immunohistochemical staining were rat anti-mouse CD11b (1:600, AbDSerotec, Hercules, CA, USA, MCA711 clone 5C6) and rabbit anti-NG2 (1:500, Chemicon, Billerica, MA, USA, AB5320). Isotype control antibodies were rabbit immunoglobulin (Ig)G (1:20,000, DakoCytomation, Carpinteria, CA, USA, X0903) and rat IgG2_b_ (1:600, Biosite, Plymouth Meeting, PA, USA, IG-851125). Visualization of CD11b^+^ microglia-macrophages was performed using the three-step biotin-streptavidin-horseradish peroxidase technique described by Lambertsen and colleagues, 2001 [14]. Visualization of NG2^+^ OPCs was performed using peroxidase-labeled “ready-to-use” EnVision^+^ polymer (K4300, DakoCytomation) according to the manufacturer’s instructions on spinal cord sections demasked using 0.5% Pepsin (Sigma-Aldrich, P-7012) in HCl and H_2_O for 10 minutes at 37°C. Sections were counterstained using Hematoxylin Gills or Toluidine blue. Isotype controls were devoid of staining (not shown).

### Estimation of the total number of CD11b^+^ and NG2^+^ cells

Using an approximated stereological counting technique unaffected by shrinkage/tissue resorption [15], we estimated the total number of CD11b^+^ and NG2^+^ cells in the spinal cord of naïve IκBα-dn and WT mice and the total number of CD11b^+^ cells in IκBα-dn and WT mice that had survived 3 days and 6 weeks after SCI. Briefly, cells with a clearly identifiable H&E or Toluidine Blue stained nucleus in conjunction with a detectable immunohistochemical signal were counted on approximately 13 sections in naïve cords and at 3 days, and on 17 sections 6 weeks after injury separated by 250 μm from each animal, using a 100× objective and a 2,470 μm^2^ frame area stepping 150 μm/150 μm in the XY-position using the CAST Grid System from Olympus (Ballerup, Denmark). The total number (N) of cells in each animal was estimated using the formula: Estimate of N = ∑Q × (1/ssf) × (1/asf) × (1/tsf), where 1/tsf is the thickness sampling fraction (1/tsf = 1), 1/ssf the sampling section fraction (1/ssf = 10), and 1/asf the area sampling fraction (22,500/2,470) as previously described [16]. In naïve mice and for the time point of 3 days we, for consistency, analyzed a total of 3.25 mm long piece of mouse spinal cord, 1.625 mm on pre- and post-epicenter. For the time point of 6 weeks we analyzed a 4.25 mm long piece of mouse spinal cord, 2.125 mm on both sides of the epicenter.

### Estimation of the lesion and white matter volumes

The lesion volume and the white matter volume were estimated on Luxol Fast Blue serial sections counterstained with H&E using the Neurolucida software (MBF Bioscience, Williston, VT, USA) as previously described [12].

### Immunofluorescent staining

For BrdU immunofluorescent staining, cryostat sections were thawed at room temperature for 5 minutes, rinsed in 1X PBS, and processed for antigen retrieval using 2N HCl for 30 minutes at 37°C. The sections were then neutralized for 10 minutes in 0.1 M sodium borate (pH 8.5) and rinsed in 1X PBS. After blocking 30 minutes in 5% BSA/5% normal goat serum (NGS)/0.3% Triton X100/PBS, rat anti-BrdU antibody (1:200, Novus Biologicals, Littleton, CO, USA; diluted in 4% BSA/3% NGS/0.1% Triton X100/PBS) was applied to the sections in combination with either mouse anti-adenomatous polyposis coli (APC; clone CC1) antibody (1:500, Calbiochem, Billerica, MA, USA) or rabbit anti-NG2 antibody (1:500, Chemicon), and incubated overnight at 4°C. For triple immunostaining we used rat anti-BrdU (1:200, Novus Biologicals) and rabbit anti-Olig2 (1:500, Millipore, Billerica, MA, USA) with either mouse anti-NG2 (1:200, Millipore) or mouse anti-APC (1:500, Calbiochem). Following extensive rinses in 1X PBS, Alexa-conjugated secondary antibodies (1:500, Molecular Probe, Grand Island, NY, USA) were applied for 30 min at room temperature. Sections were finally rinsed and mounted with Vectashield (Vector Laboratories, Burlingame, CA, USA). To estimate the number of BrdU^+^/CC1^+^, BrdU^+^/NG2^+^, and total CC1^+^-cells following SCI, serial sections were counted using Zeiss Axiovert 200M fluorescent microscope (63X objective; Thornwood, NY, USA) and Stereo Investigator software (MicroBrightField, Williston, VT, USA) for unbiased stereological estimation of cell numbers. For each section a 50 × 50 μm counting frame and a 120 × 120 μm grid was used to count the cells at 250 μm intervals. A total number of 11 sections, centered on the lesion site, were counted. For the number of CC1^+^ cells in the naïve thoracic spinal cord, a total number of 5 sections were counted.

For CXCR4 immunostaining, thawed cryostat sections were fixed and permeabilized in ice-cold acetone for 10 minutes at −20°C, then rinsed in PBS and blocked for 1 hour in 10% NGS/PBS and 30 minutes in 5% BSA/PBS. Sections were then incubated overnight with rabbit anti-CXCR4 antibody (1:500, Abcam, Cambridge, MA, USA) diluted in 5% BSA/1% NGS/PBS in combination with either mouse anti-GFAP (1:500, BD Pharmingen, San Jose, CA, USA) or mouse anti-APC (1:500, Calbiochem) antibodies. Alexa-conjugated secondary antibodies (1:500, Molecular Probes) diluted in 5% BSA/1% NGS/PBS were applied to the rinsed sections for 30 minutes at room temperature. Then sections were rinsed and mounted with Vectashield with 4',6-diamidino-2-phenylindole (DAPI) (Vector Laboratories). For toll-like receptor 4 (TLR4; 1:50, Santa Cruz, Dallas, TX, USA) and TNF receptor 2 (TNFR2; 1:200, Santa Cruz), a similar protocol was used except that the sections were permeabilized and blocked in 5% BSA/5% NGS/0.3% Triton X100/PBS. Nuclei were visualized using a DAPI counterstain. Images were obtained with an Olympus FluoView 1000 confocal microscope.

### Total RNA isolation

Total RNA was isolated from spinal cord samples (1.5 cm centered on the lesion site) using TRIzol reagent (Invitrogen, Grand Island, NY, USA) according to the manufacturer’s directions. Precautions were taken to preserve RNA integrity during the isolation, including rapid dissection on ice with RNase-free dissecting tools followed by flash-freezing in liquid nitrogen of the spinal cord segment sample as previously described by Brambilla and colleagues [6]. RNA integrity was determined with the Bioanalyzer 2100 (Agilent Technologies, Santa Clara, CA, USA).

### Microarray analysis and data processing

Microarray experiments were conducted at the University of Miami DNA and Microarray Core Facility (http://www.mihg.org/weblog/core_resources/2007/11/microarray-and-gene-expression.html) using Agilent Whole Mouse Genome Oligo microarrays (Agilent Technologies). Arrays were scanned at a 5 μm resolution using a GenePix 4000B scanner (Axon Instruments at Molecular Devices) and images analyzed with the software GenePix Pro 6.1 (Axon Instruments at Molecular Devices, LLC, Sunnyvale, CA, USA). Extracted data were transferred to the software Acuity 4.0 (Axon Instruments at Molecular Devices) for quality control. Features for further analysis were selected according to the following quality criteria: at least 90% of the pixels in the spot with intensity higher than background plus two standard deviations; less than 2% saturated pixels in the spot; signal to noise ratio (ratio of the background subtracted mean pixel intensity to standard deviation of background) 3 or above for each channel; spot diameter between 80 and 110 μm; regression coefficient of ratios of pixel intensity 0.6 or above. To identify significantly expressed genes the R software LIMMA (Bioconductor, open source software at http://www.bioconductor.org) [17] was used. “Within array” normalization was carried out with Lowess normalization and “between arrays” normalization with the “quantile” algorithm in the LIMMA package. Differential expression and false discovery rate (FDR) were assessed using a linear model and empirical Bayes moderated F statistics [18,19]. Genes with FDR below 1% were considered statistically significant. All primary microarray data were submitted to the public database at the GEO website (http://www.ncbi.nih.gov/geo; record number: GSE46695). Selected genes were classified according to Gene Ontology category “biological process” using Onto-Express [20]. Pathway analysis was performed with WebGestalt [21]. Hierachical clustering was performed using GeneSpring 10.0 (Agilent Technologies). All experiments were performed in three replicates/groups/time points.

### Quantitative real-time PCR

An aliquot of 2 μg of spinal cord RNA from each time point was reverse transcribed using the omniscript RT-PCR kit (Qiagen, Valencia, CA, USA) as previously described [6]. qPCR was performed with the Rotor-Gene 3000 Real Time Cycler (Corbett Research, Valencia, CA, USA) on cDNA samples with TAQurate GREEN Real-Time PCR MasterMix (Epicentre Biotechnologies, Madison, WI, USA) as previously described [6] for the following genes: CXCR4 (forward primer: TGT GAC CGC CTT TAC CCC GAT AGC, reverse primer: TTC TGG TGG CCC TTG GAG TGT GAC), TLR4 (forward primer: TGC CCC GCT TTC ACC TC, reverse primer: ACC AAC GGC TCT GAA TAA AGT GT), Lingo-1 (forward primer: GAC TGC CGG CTG CTG TGG GTG TT, reverse primer: CCG GCG GCA GGT GAA GTA GTT GG), Sox17 (forward primer: CGG CGC AAG CAG GTG AAG, reverse primer: GGC TCC GGG AAA GGC AGA C), CNPase (forward primer: AGA TGG TGT CCG CTG ATGCTT AC, reverse primer: CTC CCG CTC GTG GTT GGT), CD11b (forward primer: GCC CCA AGA AAG TAG CAA GGA GTG, reverse primer: TAC GTG AGC GGC CAG GGT CTA AAG) and ICAM1 (forward primer: TGA GCG AGA TCG GGG AGG ACA G, reverse primer: GTG GCA GCG CAG GGT GAG GT). Relative expression was calculated by comparison with a standard curve after normalization to β-actin [6].

### Western blotting

Spinal cords (1.5 cm centered on the injury site) were homogenized in 300 μl radio immunoprecipitation assay buffer (0.01 M sodium phosphate pH 7.2, 0.15 M NaCl, 1% NP40, 1% sodium deoxycholate, 0.1% SDS, 2 mM EDTA) supplemented with complete protease inhibitor cocktail (Roche, Indianapolis, IN, USA), incubated for 30 minutes at 4°C on an end-over-end rotator, and centrifuged at 4°C for 10 minutes at 14,000 rpm. The supernatant was then transferred to a fresh tube on ice and an aliquot was used for protein quantification using the *DC* Protein Assay (Biorad, Hercules, CA, USA). Equal amounts of proteins were resolved by SDS-PAGE on 10% or 15% gels, transferred to nitrocellulose membranes, and blocked in 5% nonfat milk in 0.1 M Tris buffered saline-triton (TBS-T) for 1 hour at room temperature. Membranes were probed with an antibody recognizing either proteolipid protein (PLP; mouse monoclonal, Millipore, 1:250), CXCR4 (rabbit polyclonal, Abcam, 1:500), Foxc2 (mouse monoclonal, Santa Cruz, 1:500), TLR4 (mouse monoclonal, Santa Cruz, 1:200), TNFR2 (rabbit polyclonal, Santa Cruz, 1:200), CXCR7 (rabbit polyclonal, GeneTex, Irvine, CA, USA, 1:1000) followed by horseradish peroxidase–conjugated secondary antibody (GE Healthcare, Little Chalfont, Buckinghamshire, UK, 1:2000). Proteins were visualized with a chemiluminescent kit (ECL; GE Healthcare). Blots were also probed for β-actin (mouse monoclonal, Santa Cruz, 1:500) as a loading control. The data were analyzed using Quantity One software (Biorad).

### Data analysis

One-way or two-way analysis of variance (ANOVA) followed by the appropriate *post hoc* test and Student’s *t*-test (one-tailed and two-tailed). Statistical analyses were performed using Prism 4.0b software for Macintosh, GraphPad Software, San Diego, CA, USA, http://www.graphpad.com. Data are presented as mean ± SEM. Statistical significance was established for *P* < 0.05.

## Results

### Oligodendrogenesis is increased following spinal cord injury in mice lacking functional NF-κB signaling in astrocytes

**Figure 1:**
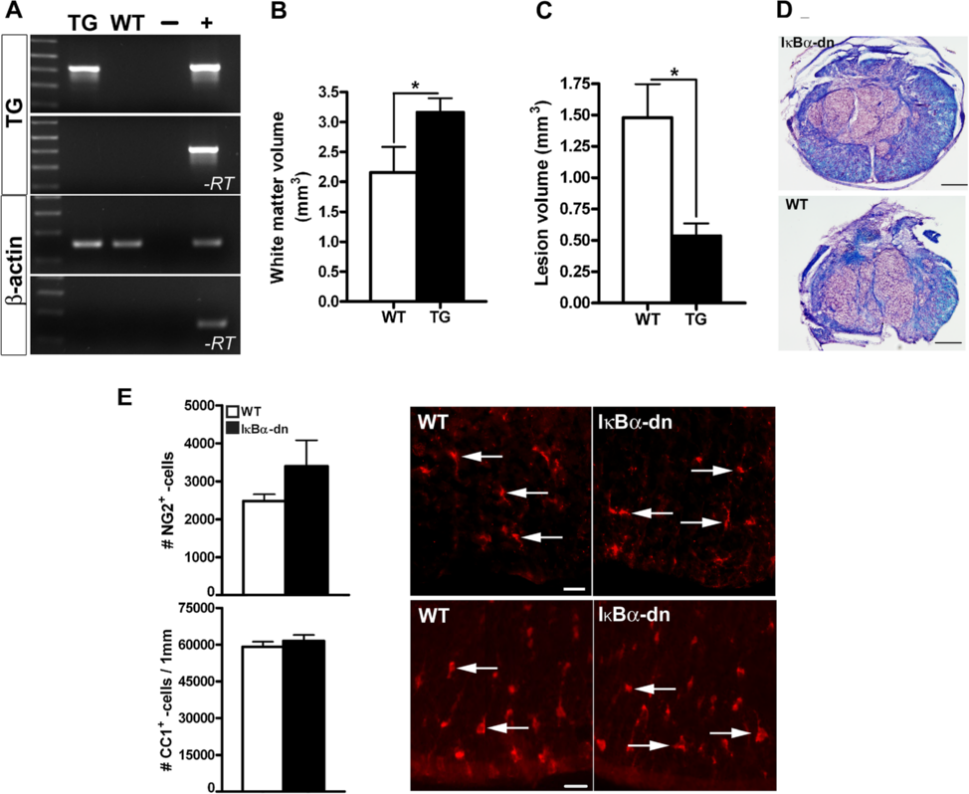
**Inhibition of astroglial NF-κB does not affect the number of oligodendrocyte precursor cells and mature oligodendrocytes in the naïve, murine adult spinal cord. (A)** IκBα-dn transgene (TG) verification in GFAP-IκBα-dn (TG) mice. Total RNA was isolated from the spinal cord and RT-PCR performed with primers to the TG or β-actin as control. Controls for genomic DNA contamination, where the reverse transcriptase is omitted (−*RT*) were included as well as negative (−, water) and positive (+, genomic DNA) controls for the PCR reaction. **(B)** Estimation of white matter volume 6 weeks post-injury was performed on Luxol Fast Blue sections counterstained with H&E and showed increased white matter volume in IκBα-dn TG mice compared to wild-type (WT) mice. **(C)** Estimation of the lesion volume showed significantly decreased mean lesion volume in IκBα-dn TG mice compared to WT mice. **(D)** Representative Luxol-stained sections from GFAP-IκBα-dn and WT littermates. Scale bar = 350 μm. **(E)** Estimation of the total number of oligodendrocyte precursor cells (OPCs) using the nerve/glial antigen 2 (NG2) marker and the total number of mature oligodendrocytes using the adenomatous polyposis coli marker (CC1) showed similar numbers of OPCs and mature oligodendrocytes in naïve IκBα-dn TG and WT mice. Immunohistochemistry using the NG2 antibody showed that NG2^+^ OPCs were distributed evenly throughout the white matter in both WT and IκBα-dn mice. Representative immunohistochemistry using the CC1 antibody showed that CC1^+^ oligodendrocytes were distributed evenly throughout the white matter in both WT (left) and IκBα-dn (right) mice. Scale bar = 20 μm. N = 4 to 5 animals per group, Student’s *t*- test (one/two-tailed).

Based on our previous findings of a reduced lesion volume, increased white matter preservation and associated improvements in locomotor function 8 weeks following moderate contusion to the thoracic spinal cord in mice lacking astroglial NF-κB [12], we wanted to investigate the possibility that the observed increase in white matter is due, in part, to enhanced oligodendrogenesis. Since our GFAP-IκBα-dn mice were generated 7 years ago and may have been affected by genetic drift over time, we decided to confirm by RT-PCR that the transgene (IκBα-dn) was indeed still expressed in the spinal cord of our transgenic mice (Figure 1A). We also confirmed that, 6 weeks following SCI, GFAP-IκBα-dn mice displayed a significantly smaller lesion volume, associated with a significantly larger white matter volume (Figure 1B-D). This was also reflected by a significant improvement of locomotor performance in the open field test, scored by the basso mouse scale [22] (IκBα-dn: 5.4 vs WT: 4.1, *P* < 0.05). Next, we investigated whether there were any abnormalities in the morphology of the spinal cord and in the total number of OPCs and mature oligodendrocytes, due to expression of the IκBα-dn transgene in astrocytes. In order to do so, total numbers of NG2^+^ OPCs (Figure 1E, upper panel) and CC1^+^ oligodendrocytes (Figure 1E, lower panel) were estimated in spinal cord sections from naïve WT and IκBα-dn mice. We found that the spinal cords from naïve WT and IκBα-dn mice appeared morphologically identical [12] and displayed similar numbers of NG2^+^ OPCs (WT: 2,479 ± 181; IκBα-dn: 3,397 ± 683, *P* = 0.23) and CC1^+^ oligodendrocytes (WT: 59,190 ± 2,086; IκBα-dn: 61,540 ± 2,447, *P* = 0.504) (Figure 1E).

**Figure 2:**
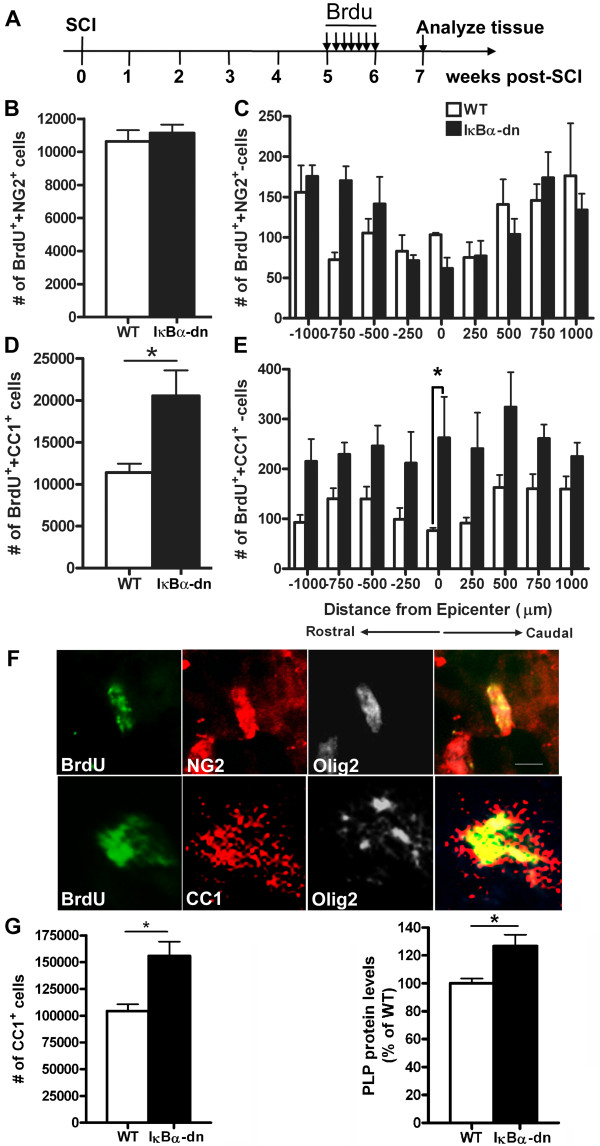
**Oligodendrogenesis is increased in IκBα-dn mice lacking functional NF-κB signaling in astrocytes. (A)** Mice were subjected to moderate spinal cord contusion at T9 and received bromodeoxyuridine (BrdU) injections once a day for 1 week starting 5 weeks post-injury. Spinal cord tissue (in total 2 mm centered on injury) was analyzed 7 weeks post-spinal cord injury (SCI). **(B**, **C)** The total estimated number of BrdU^+^NG2^+^ cells using Stereo Investigator software in a 2-mm segment of spinal cord centered on the site of injury was similar between wild-type (WT) and IκBα-dn mice **(B)** with a similar distribution over the injured spinal cord **(C)**. **(D**, **E)** In contrast, the total estimated number of BrdU^+^CC1^+^ cells was significantly increased in IκBα-dn mice (**D**, *P < 0.05, Student’s *t-*test) with a higher number of newly formed oligodendrocytes around the epicenter compared to those in WT mice (**E**, two-way analysis of variance; *P<0.05 Bonferroni post-test). **(F)** Representative pictures of BrdU^+^NG2^+^ and Brdu^+^CC1^+^ cells showing co-labeling with the oligodendroglial lineage marker Olig2. **(G**, left**)** At this time point, the total number of mature oligodendrocyte (CC1^+^ cells) in the injured spinal cord of IκBα-dn mice was also significantly (*P < 0.05, Student’s *t-*test) higher than in WT mice. **(G**, right**)** Western blot quantification on mice with 6 weeks survival also showed a significant increase in the myelin protein PLP in IκBα-dn mice compared to WT mice (*P < 0.05, Student’s *t-*test) supporting increased oligodendrogenesis in IκBα-dn mice already at 6 weeks post-SCI. N = 4 animals per group. NG2, nerve/glial antigen 2.

In order to investigate changes in oligodendrogenesis following SCI, we administered BrdU daily for 7 days starting the fifth week following injury and sacrificed the mice 2 weeks later (7 weeks post-SCI) so that the BrdU-labeled OPCs had time to differentiate into mature oligodendrocytes [2] (Figure 2A). To investigate changes in numbers of newly formed OPCs and newly formed mature oligodendrocytes, we performed double immunostaining for BrdU, and NG2 or CC1, respectively, and estimated the total number of BrdU^+^NG2^+^ and BrdU^+^CC1^+^ cells in 2-mm long spinal cord segments 7 weeks after SCI. We found no significant difference in the number of BrdU^+^NG2^+^ cells between IκBα-dn mice (11,140 ± 503) and WT mice (10,640 ± 679) (*P* = 0.57) (Figure 2B,C). However, we did find a significant increase in the number of BrdU^+^CC1^+^ cells in the injured spinal cord of IκBα-dn mice (20,550 ± 3,043) compared to that of WT mice (11,400 ± 1,062) (Figure 2D, *P* < 0.05) suggesting that blocking astroglial NF-κB promotes oligodendrogenesis. Furthermore, when looking at the distribution of the BrdU^+^CC1^+^ cells rostrally and caudally from the epicenter, we found significantly more BrdU^+^CC1^+^ cells around the epicenter in the IκBα-dn mice compared to WT mice, suggesting that the microenvironment within or near the lesion core, in the IκBα-dn mice, is more permissive for differentiation of OPCs into mature oligodendrocytes (Figure 2E). Triple immunofluorescence staining confirmed that BrdU^+^NG2^+^ and BrdU^+^CC1^+^ cells colocalized with Olig2^+^ cells, another marker for OPCs and mature oligodendrocytes [23] (Figure 2F). To further confirm increased oligodendrogenesis in the IκBα-dn mice, we estimated the total number of mature CC1^+^ oligodendrocytes in 2-mm long spinal cord segments 7 weeks after SCI. Supporting our finding of increasing numbers of mature BrdU^+^CC1^+^ oligodendrocytes in IκBα-dn mice (Figure 2D), we found significantly more CC1^+^ cells (*P* = 0.04) in the injured spinal cord of IκBα-dn mice (155,800 ± 13,490) compared to injured WT spinal cord (104,300 ± 6,356) 7 weeks after SCI (Figure 2G, left). These data were furthermore supported by findings of significantly increased PLP protein levels in the spinal cords of IκBα-dn mice 6 weeks after injury compared to injured WT mice (Figure 2G, right), which further points to an increased oligodendrogenesis after SCI in IκBα-dn mice. Collectively, these data demonstrate that inhibiting astroglial NF-κB enhances oligodendrogenesis following SCI.

### Microarray analysis of the spinal cord from wild-type and IκBα-dn mice following spinal cord injury

**Figure 3:**
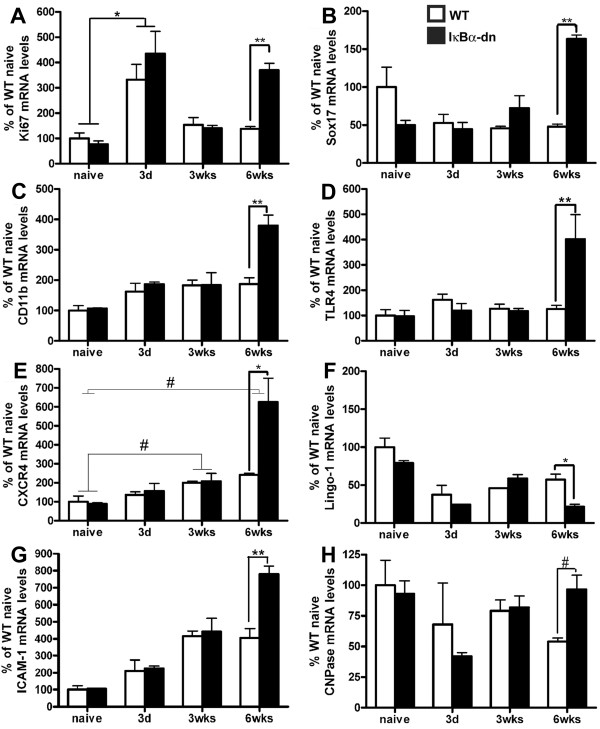
**Quantitative real-time PCR was used to confirm a number of differentially regulated genes between wild-type (WT) and IκBα-dn mice.** *P < 0.05, **P < 0.01, two-way analysis of variance followed by Bonferroni post tests; ^#^*P* < 0.05, *t*-test; N = 3 animals per group. 3d, 3 days; 3 wks, 3 weeks; 6 wks, 6 weeks.

To elucidate the molecular mechanisms leading to the observed increased oligodendrogenesis, we compared gene expression profiles using Whole Mouse Genome microarrays, which included 41,000 genes and transcripts from naïve and injured WT and IκBα-dn mice. The experiments were performed using three biological replicates per group using naïve animals as well as three different survival times - 3 days, 3 and 6 weeks post-SCI. We concentrated on genes with a fold-change greater than 2.0 and a FDR <0.1%. We identified 66 differentially expressed genes between naïve mice, 35 genes were differentially expressed 3 days after SCI, 108 genes were differentially expressed at 3 weeks and at 6 weeks 994 genes were found to be differentially expressed (Table 1). Significant changes were especially present 6 weeks after SCI in genes involved in inflammatory/immune responses, chemotaxis, motor axon guidance, axonal growth, cell death, signal transduction, and so on, all processes that may influence functional recovery. For a functional classification of a subset of transcripts 6 weeks after SCI please refer to Table 2 and The National Center for Biotechnology Information Gene Expression Omnibus GSE46695 for a list of all transcripts. Relative transcript enrichment detected by microarrays was confirmed by qPCR for eight genes (Ki67, Sox17, CD11b, TLR4, CXCR4, Lingo-1, ICAM1 and CNPase) selected from the 6 weeks gene groups (Figure 3A-H).

**Table 1:** Microarray data summary

**Time post-SCI**	**Total number of differentially expressed genes**	**Genes under expressed in GFAP-IκBα-dn mice**	**Genes over expressed in GFAP-IκBα-dn mice**
**Naive**	66	15 (22.7%)	51 (77.3%)
**3 days**	35	3 (8.6%)	32 (91.4%)
**3 weeks**	108	69 (63.9%)	39 (36.1%)
**6 weeks**	994	596 (60.0%)	398 (40.0%)

Number of differentially expressed genes between wild-type and GFAP-IκBα-dn mice at various time points following spinal cord injury (SCI). Results are derived from the analysis of three biological replicates/time points.

**Table 2:** Genes associated directly or indirectly with myelination

**Gene name**	**Accession number**	**Fold change**
		**GFAP-IκBα-dn mice versus wild-type mice at 6 weeks**
*Chemokine-Chemokine receptors*		
Cxcl12	NM_013655	+ 2.19
Cxcr4	NM_009911	+ 2.01
*Transcription factors*		
Foxc2	NM_013519	+ 6.55
Sox17	NM_0011441	+ 5.35
Tcf4	NM_009333	+ 2.24
*Proliferation marker*		
mKi67	X82786	+ 2.43
*Microglia/leukocytes*		
Itga1 (CD11b)	NM_176922	+ 2.03
CD200r	NM_021325	+ 2.49
TLR4	NM_021297	+ 2.38
*Inhibitor*		
Lingo1	BC008626	- 2.07
*Myelin*		
PMP2	NM_001030305	+ 2.33

Thus far we have presented data suggesting that inhibiting NF-κB activation in astrocytes promotes an environment favorable for oligodendrogenesis (Figures 2 and 3). To explore oligodendrogenesis further, we focused on genes previously demonstrated to be important in cell proliferation and oligodendrogenesis such as Sox17 and Lingo-1 [24,25]. While not a specific indicator of oligodendrogenesis, we found that Ki67, a general marker of proliferation, was significantly elevated 3 days post-SCI in both WT and IκBα-dn mice relative to naïve animals but only in IκBα-dn mice 6 weeks post-SCI (Figure 3A). Some possible sources for Ki67 expression, besides infiltrating immune cells, are also OPCs. Sox17, a transcription factor important in oligodendrocyte development [26], was significantly upregulated in IκBα-dn mice 6 weeks post-SCI (Figure 3B), while Lingo-1, a negative regulator of oligodendrogenesis [27], was significantly reduced in IκBα-dn mice at this time point (Figure 3F). These findings support the data presented in Figure 2 showing significantly increased numbers of BrdU^+^CC1^+^ oligodendrocytes, significantly increased numbers of CC1^+^ oligodendrocytes and significantly increased PLP levels in IκBα-dn mice, suggesting increased oligodendrogenesis in the IκBα-dn mice compared to WT mice.

### Inhibition of astroglial NF-κB results in an altered inflammatory state that is supportive of oligodendrogenesis after spinal cord injury

**Figure 4:**
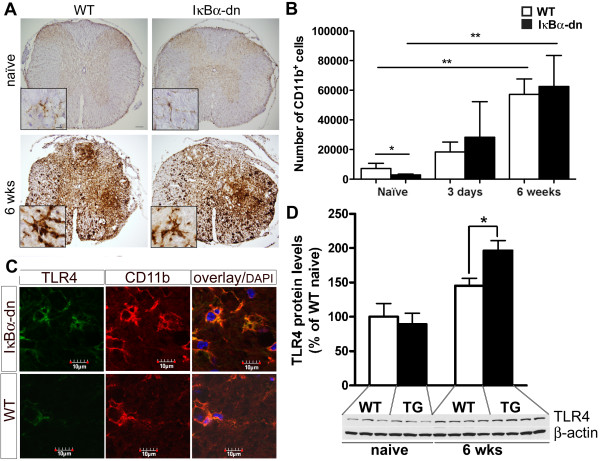
**Quantification of microglia/leukocytes in the naïve, 3 days, and 6 weeks injured spinal cord. (A)** Representative immunohistochemical staining for CD11b in naïve wild-type (WT) and IκBα-dn mice and 6 weeks (wks) following spinal cord injury (SCI). **(B)** The total number of CD11b^+^ cells were significantly increased in naïve IκBα-dn mice compared to WT mice and significantly increased 6 weeks after SCI in both IκBα-dn and WT mice. Each bar represents the average cell count ± SEM. *P < 0.05, **P < 0.01, N = 4 to 9 animals per group. **(C)** Representative photomicrographs of immunohistochemical stainings for toll-like receptor 4 (TLR4) in the injured spinal cord white matter of WT and IκBα-dn mice, showing a robust staining on CD11b^+^ microglia/leukocytes from the chronically injured IκBα-dn mice. **(D)** Western blot quantification showing a significant increase in TLR4 in IκBα-dn mice (TG) compared to WT mice 6 weeks after SCI. N = 3 to 4 animals per group, *P < 0.05. DAPI, 4',6-diamidino-2-phenylindole.

An inflammatory reaction following traumatic injury is necessary to contain the injury and clear debris, and microglia - the resident macrophages of the central nervous system (CNS) - are rapidly activated following disturbances and secrete pro-inflammatory cytokines [28,29]. Different phenotypes of microglia have been identified [30] and even though often associated with neuroinflammatory processes, their role has been extended to maintenance and repair of the nervous tissue where they reside [31,32], some of them being supportive of remyelination [33,34]. Also, distinct subsets of macrophages have been shown to cause either toxicity or regeneration in the injured mouse spinal cord [35]. Since in the present study we found a significant increase in CD11b mRNA levels using qPCR in our IκBα-dn mice compared to WT mice at 6 weeks post-SCI (Figure 3C), we further estimated the total number of CD11b^+^ microglia/leukocytes (Figure 4A, shown for naïve and 6 weeks). In naïve mice there were significantly more CD11b^+^ cells in WT mice compared to IκBα-dn mice (*P* < 0.05, Figure 4B). However, counting CD11b^+^ microglia/leukocytes in both IκBα-dn and WT mice 3 days and 6 weeks after SCI did not show evidence of a difference in the total number of CD11b^+^ cells between the two genotypes, even though the total number of CD11b^+^ cells was significantly increased in both IκBα-dn and WT mice 6 weeks after SCI compared to naïve mice (*P* < 0.001, one-way ANOVA) (Figure 4A,B). These data suggest that the microglial numbers and leukocyte infiltration is similar between IκBα-dn and WT mice but that the transcriptional regulation of CD11b mRNA levels and possibly the activation status of these cells 6 weeks after SCI are differently regulated in IκBα-dn mice compared to WT mice.

Since TLR4, a pattern recognition receptor important in innate immunity that has been shown to modulate myelination, astrogliosis and macrophage activation [34,36], was found to be up-regulated in the microarray at 6 weeks post-injury in the IκBα-dn mice, we confirmed by qPCR the significant increase in TLR4 mRNA in IκBα-dn mice (Figure 3D). We further examined the cellular expression of TLR4 in injured spinal cord tissue from WT and IκBα-dn mice by immunohistochemistry. TLR4 immunoreactivity colocalized almost exclusively with CD11b^+^ microglia/leukocytes in both WT and IκBα-dn mice and showed stronger immunoreactivity in the injured spinal cord of the IκBα-dn mice compared to WT (Figure 4C), suggesting a difference in the state of activation of microglia/leukocytes between the two genotypes. This was further supported by the finding of a significant increase in TLR4 protein levels in IκBα-dn mice 6 weeks post-SCI compared to WT LM (*P* < 0.05, Figure 4D).

### CXCR4 expression is increased on oligodendrocytes following spinal cord injury

**Figure 5:**
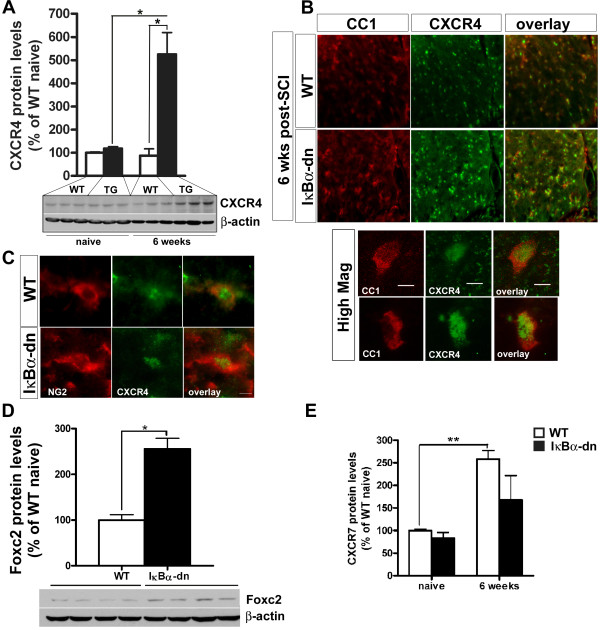
**CXCR4 expression is increased on oligodendrocytes in IκBα-dn mice. (A)** Western blot analysis of naïve and injured spinal cords showed a significant increase in CXCR4 protein levels in IκBα-dn mice compared to wild-type (WT) mice 6 weeks post-spinal cord injury (SCI). **(B)** Representative photomicrographs of mature CC1^+^CXCR4^+^ oligodendrocytes in IκBα-dn and WT spinal cord 6 weeks after SCI. **(C)** Representative photomicrograph of a NG2^+^CXCR4^+^ in WT and IκBα-dn spinal cord 6 weeks post-SCI. **(D)** Western blot analysis for Foxc2 shows significantly increased expression in IκBα-dn mice compared to WT mice 6 weeks post-SCI. **(E)** CXCR7 expression following SCI, examined by Western blot analysis, showed that CXCR7 levels were significantly increased only in WT mice 6 weeks after SCI, whereas no significant increase was observed in IκBα-dn mice. Each bar represents mean ± SEM. N = 3 to 4 animals per group. *P < 0.05 and **P < 0.01. NG2, nerve/glial antigen 2.

Chemokines and their receptors are also known to be important regulators of inflammation and repair processes following CNS injury [37]. Signaling through the alpha chemokine receptor CXCR4 is required for migration of neuronal precursors, axon guidance/pathfinding, neurite growth and maintenance of neuronal progenitor cells as well as oligodendrocyte progenitors and remyelination [38-42]. Furthermore, CXCL12 signaling through CXCR4 enhances the infiltration of monocytes and lymphocytes in different inflammation models [43,44]. In line with these findings, CXCR4 mRNA levels were significantly upregulated in IκBα-dn and WT mice at 3 and 6 weeks after SCI compared to naïve mice (Figure 3E). Furthermore, at 6 weeks post-SCI, IκBα-dn mice displayed significantly higher CXCR4 mRNA levels compared to injured WT mice (Figure 3E). This was further confirmed using western blotting and immunohistochemical expression analysis of CXCR4 (Figure 5A,B). In line with qPCR analysis, CXCR4 protein levels were significantly upregulated in IκBα-dn mice 6 weeks after SCI compared to naïve mice, (*P* = 0.013) and compared to WT mice with 6 weeks survival (*P* = 0.038, Figure 5A). CXCR4 was expressed in CC1^+^ oligodendrocytes both in IκBα-dn and WT mice with increased expression in IκBα-dn mice (Figure 5B, shown for 6 weeks). CXCR4 was also found to be expressed in some NG2^+^-cells 6 weeks after SCI (Figure 5C).

Since the transcription factor Foxc2 is important in CXCR4 regulation [45], we further compared Foxc2 expression at this time point using western blotting. In line with the findings of significantly increased protein CXCR4 levels in IκBα-dn compared to WT mice, Foxc2 protein levels were also significantly upregulated 6 weeks post-SCI in IκBα-dn compared to WT mice (Figure 5D).

Furthermore, CXCR7 has been implicated in the pathophysiology of demyelination and axonal injury in EAE where antagonism of CXCR7 promotes functional recovery and reduces axonal injury [46]. CXCR7 was not in the microarray analysis but based upon the role it plays in EAE and our present results on CXCR4, we investigated CXCR7 protein expression following SCI (Figure 5E). We detected a significant increase in CXCR7 expression 6 weeks post-SCI in WT mice but not in IκBα-dn mice compared to naïve spinal cords (Figure 5E), suggesting that CXCR7 expression is significantly reduced by inhibition of NF-κB in astrocytes.

### IκBα-dn mice displayed increased TNFR2 expression compared to wild-type mice after spinal cord injury

**Figure 6:**
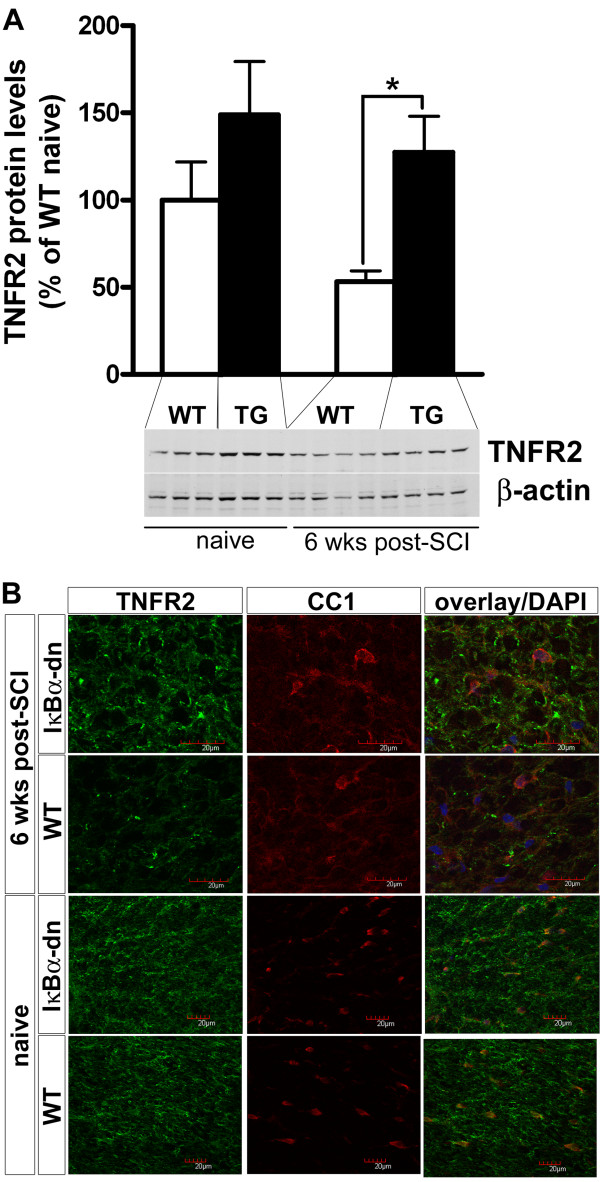
**TNFR2 expression is increased in the IκBα-dn injured spinal cord compared to wild-type 6 weeks post-spinal cord injury. (A)** Quantification of TNFR2 protein expression levels in the injured spinal cord of wild-type (WT) and IκBα-dn mice showing significantly less protein in WT mice 6 weeks after spinal cord injury (SCI) compared to IκBα-dn mice. Each bar represents the mean ± SEM. *P < 0.05. N = 3 to 4 animals per group. **(B)** Representative confocal images of mature CC1 oligodendrocytes and TNFR2 expression in naïve and injured spinal cord from WT and IκBα-dn mice, showing that a subset of TNFR2^+^ cells co-localizes with CC1^+^ oligodendrocytes.

TNF signaling through TNFR2 has been shown to promote proliferation of OPCs and remyelination [47] and recently, using XPro1595 a specific inhibitor for soluble TNF in EAE, we demonstrated a beneficial role of TNFR2 signaling on functional outcome [48]. Based upon these data, we sought to determine what effect inhibiting astroglial NF-κB would have on TNFR2 expression following SCI. As shown in Figure 6A, there was significantly increased TNFR2 protein expression in IκBα-dn mice 6 weeks after injury compared to injured WT mice (Figure 6A), due to a decrease in protein levels in WT mice that did not occur in IκBα-dn mice. These findings were supported by immunohistochemical stainings showing increased levels of TNFR2 in WT mice compared to IκBα-dn mice, whereas the levels in naïve spinal cords appeared similar (Figure 6B). In naïve spinal cords, TNFR2 was expressed primarily by oligodendrocytes (Figure 6B) whereas 6 weeks after SCI, TNFR2 expression was also expressed in other types of cells (Figure 6B), probably microglia and infiltrating macrophages, as shown previously for other CNS injuries [49]. These data, along with our previous studies, suggest that enhanced oligodendrogenesis could be due in part to the sustained expression of TNFR2 in IκBα-dn mice following injury.

Collectively, our data suggest that sustained expression of TNFR2 in IκBα-dn mice enhances the expression of CXCR4 and thereby promotes an environment supportive of oligodendrogenesis and remyelination. Furthermore, CXCR7 is expressed on astrocytes and signals through NF-κB suggesting that the reduced neuropathology in our IκBα-dn mice could be due to impaired CXCR7 expression and signaling in these mice.

## Discussion

In a previous study, we showed that mice lacking functional NF-κB signaling in astrocytes (GFAP-IκBα-dn transgenic mice) recover better following moderate spinal cord contusion, with a significant improvement in locomotor function that correlates with a smaller lesion area and a larger area of white matter preservation compared to injured WT LM [12]. Since the mice were generated several years ago, we confirmed that they still retained the same phenotype following SCI and found that the GFAP-Iκbα-dn mice still did perform significantly better than the WT mice on the Basso Mouse Scale following moderate SCI, supported by a smaller lesion size and more myelin 6 weeks post-SCI. The larger white matter volume in the IκBα-dn transgenic mice could be due to sparing of oligodendrocytes from cell death and/or to an increase in oligodendrogenesis. In the present paper, we demonstrate that there is indeed an increase in oligodendrogenesis in the IκBα-dn transgenic mice compared to the WT LM 6 to 7 weeks post-SCI. In the naïve mice we did not find any differences in the number of oligodendrocytes or the amount of myelin between WT and transgenic mice suggesting that blocking NF-κB signaling in astrocytes under naïve conditions does not affect myelination. However, following SCI we found a large increase in the number of newly formed BrdU^+^/CC1^+^ oligodendrocytes, suggesting that astroglial NF-κB directly or indirectly affects the differentiation of OPC into mature, myelinating oligodendrocytes. In fact, a recent study showed that reactive astrocytes from the injured spinal cord can inhibit oligodendrocyte differentiation in vitro [11]. Many others have also shown that astrocytes can directly modulate myelination in vitro via the release of a number of secreted factors, depending on culture conditions [7,10]. In our study, it is so far unknown whether the decreased expression of a NF-κB-regulated gene has a direct effect on oligodendrocyte maturation or an indirect effect through other cells such as microglia and/or infiltrating macrophages. Indeed, factors secreted by macrophages from the injured spinal cord have been shown to inhibit growth of NG2^+^ cells in vitro [50]. When we examined the distribution of the BrdU^+^/CC1^+^ cells, we found numerous double immunolabeled cells located around the epicenter in the IκBα-dn transgenic mice compared to the WT LM mice, suggesting that while the lesion epicenter in the WT mice is inhibitory for oligodendrocyte differentiation, the epicenter environment in the transgenic mice is more permissive and may allow for a better survival of newly generated oligodendrocytes. To gain insight into the molecular mechanisms underlying increased oligodendrogenesis, we performed a microarray analysis on naïve and injured spinal cords at 3 days, 3 and 6 weeks post-SCI and found the largest number of differentially regulated genes between WT and GFAP-IκBα-dn mice at the more chronic time point. Since analysis of the set of genes pointed to differences in the inflammatory response with upregulation of genes such as CD11b, TLR4, CXCL12, and CXCR4 in IκBα-dn mice compared to the WT mice, we sought to determine whether differences in the number of microglia/leukocytes could account for the observed differences. Even though we did not find any differences in terms of total number of CD11b^+^ cells between WT and GFAP-IκBα-dn mice 6 weeks following SCI, we found differences in TLR4 levels, with the microglia/leukocytes from the transgenic mice showing enhanced immunoreactivity compared to the WT mice. Inflammatory reactions are important in stimulating recruitment of OPCs to demyelinating areas and in the remyelination process itself [34,51,52]. Recently, diverse microglia/macrophage phenotypes have been identified through their expression of specific sets of genes, making them either neuroprotective and reparative or toxic to the neural cells [20,33,35]. Our data suggest that inhibiting astroglial NF-κB affects the activation status of microglia/leukocytes rendering them more supportive for remyelination. In fact the role of astrocytes in modulating microglia has been highlighted in a study where astrocytes from glioblastoma have been shown to suppress microglial function [53]. It appears that the number of astrocytes may also be an important factor in the regulation of microglial function. However, we did not find any significant difference in the number of astrocytes in the spinal cord from naïve WT and IκBα-dn transgenic mice (Additional file 1).

Chemokines are essential for trafficking of leukocytes in both physiological and pathological conditions [54]. CXCL12, also known as stromal-derived growth factor 1 or SDF-1, can act through two G-coupled receptors, CXCR4 and CXCR7. CXCL12 and its receptor CXCR4 play multiple roles both in the immune and nervous systems. CXCL12 is a highly efficacious chemoattractant for lymphocytes and monocytes but not neutrophils [44]. CXCR4 signaling is required for the migration of neuronal precursors, axon guidance/pathfinding and maintenance of neural progenitor cells. In the mature CNS, CXCL12 modulates neurotransmission, neurotoxicity and neuroglial interactions. It activates NF-κB, stimulates the production of chemokines and cytokines and induces cell death in primary astrocytes [55]. CXCL12 stimulates neurite growth on inhibitory CNS myelin [40]. Regarding the role of CXCL12 in remyelination there are some divergent results as whether it promotes oligodendrocyte maturation through its receptor CXCR4 [56] or CXCR7 [57]. These apparent discrepancies may be due to the injury paradigm being a drug-induced demyelination of the corpus callosum in the study by Gottle and colleagues [57] and a myelin oligodendrocyte glycoprotein-induced EAE model in the study by Patel and colleagues [56]. In our chronically spinal cord injured mouse model, we observed a strong induction of CXCR4 on oligodendrocytes especially in IκBα-dn mice compared to WT, which is in stark contrast with the study from Gottle and colleagues [57] where they did not observe any expression of CXCR4 on oligodendroglial cells in both healthy and diseased spinal cord. The pathophysiology of SCI and EAE is very distinct, which could explain some of the differences observed in models of SCI and EAE. The pattern of expression of CXCR4 appeared mostly nuclear although we also found cytoplamic/membrane staining as well. Nuclear localization of CXCR4 has been reported following binding to CXCL12 and its function in the nucleus is still speculative [58]. As in the study by Patel and colleagues [56], we found CXCR4 expressed by some NG2^+^ cells both in WT and IκBα-dn mice. Due to the expression of CXCR4 on NG2^+^ cells and in oligodendrocytes, this would suggest a role in both myelination and oligodendrocyte survival. Regarding CXCR7, we observed an induction at 6 weeks in the injured WT mice, but not in the IκBα-dn mice while antagonism to CXCR7 has been reported to promote oligodendrocyte maturation [57] and to prevent axonal injury [46] in two different models of EAE.

TNF is a cytokine that plays different roles depending on the receptor it engages, being either TNFR1 or TNFR2. Originally viewed as a pro-inflammatory cytokine, knockout studies have demonstrated that TNF does not only have deleterious effects following CNS trauma or disease [29,59], but is also involved in the repair phase specifically through its cognate TNFR2 (p75) receptor [47]. Recently, our laboratory has demonstrated, using a specific inhibitor of soluble TNF, that signaling of membrane-bound TNF through its receptor TNFR2 was associated with axonal preservation and improved myelin compaction following EAE [48]. Furthermore a recent study by Patel and colleagues showed that TNFR2 was required for OPC proliferation and differentiation in a drug-induced demyelination model of the corpus callosum [60]. Therefore, we sought to determine whether TNFR2 expression was altered following SCI. Our data showed that TNFR2 expression was reduced 6 weeks following SCI in WT mice but was maintained at levels similar to the naïve conditions in our transgenic mice suggesting that signaling through TNFR2 on oligodendrocytes may have a positive effect on myelination as seen in EAE.

The fact that in the present study we observed dramatic gene changes at the more chronic time point may be explained by the biphasic infiltration of leukocytes in mice following SCI with a late peak occurring in the chronic phase 42 days after injury [61-63].

## Conclusion

In conclusion, our data demonstrate that one of the beneficial roles of blocking NF-κB in astrocytes is to promote oligodendrogenesis through alteration of the inflammatory environment at and around the lesion site. In particular, our data suggest that astrocytes may be modulating microglial/leukocyte activation towards a phenotype that is supportive of oligodendrogenesis and repair.

## Abbreviations

ANOVA: Analysis of variance; APC: Adenomatous polyposis coli; asf: Area sampling fraction; BrdU: Bromodeoxyuridine; BSA: Bovine serum albumin; CNS: Central nervous system; DAPI: 4',6-diamidino-2-phenylindole; EAE: Experimental autoimmune encephalomyelitis; FDR: False discovery rate; GFAP: Glial fibrillary acidic protein; H&E: Hmatoxylin and eosin; Ig: Immunoglobulin; i.p.: Intraperitoneally; LM: Littermates; NG2: Nerve/glial antigen 2; NGS: Normal goat serum; NF-κB: Nuclear factor-kappa B; OPC: Oligodendrocyte precursor cell; PBS: Phosphate buffered saline; PCR: Polymerase chain reaction; PFA: Paraformaldehyde; PLP: Proteolipid protein; RT-PCR: Reverse transcriptase-polymerase chain reaction; s.c.: Cubcutaneously; SCI: Spinal cord injury; ssf: Sampling section fraction; TBS-T: Tris buffered saline-triton; TLR: Toll-like receptor; TNF: Tumor necrosis factor; TNFR: Tumor necrosis factor receptor; tsf: Thickness sampling fraction; WT: Wild-type.

## Competing interests

The authors declare that they have no competing interests.

## Authors’ contributions

VBR participated in study design, performed the BrdU experiments, performed the immunohistochemical, biochemical and qPCR experiments and wrote the manuscript. KLL participated in study design, performed assessment of the mice, the microglia analysis, and participated in writing the manuscript. JR performed surgeries. LN performed the microarray experiment and analysis. SK participated in mouse behavioral assessment. JJ performed some oligodendrocyte counting. DGE performed the microglia analysis. BF participated in the microglia analysis, lesion volume and white matter quantification. DMM conceived the oligodendrogenesis study. JRB conceived the study and helped draft the paper. All authors read and approved the final manuscript.

## Supplementary Material

Additional file 1 **Inhibition of astroglial NF-κB does not affect the number of astrocytes in the naïve, murine adult spinal cord.** (A) Representative immunostained spinal cord cross sections from naïve wild-type (WT) and IκBα-dn transgenic (TG) mice. Astrocytes were immunostained using a polyclonal rabbit anti-GFAP (DAKO, 1:1000) and an Alexa594 anti-rabbit secondary antibody (Molecular Probe, 1:500). Hoechst was used to label the nuclei. Scale bar: 100 μm. (B) High magnification of astrocytes in the white matter spinal cord of WT and IκBα-dn TG mice. Scale bar: 20 μm. (C) Estimation of the number of astrocytes in the white matter and grey matter of a 1-mm long spinal cord segment in the thoracic region of naïve WT and IκBα-dn mice using unbiased stereology (grid size 120 μm × 120 μm and probe size 40 μm × 40 μm) showed no difference between genotypes (mean ± SEM, N = 3 per group). (D) Glial fibrillary acidic protein (GFAP) gene expression level in the spinal cord was assessed by real-time PCR. Data were normalized to β-actin and expressed as percent of WT (mean ± SEM, N = 5 per group). Click here for file
