# Supplementary figures and images for: Inhibition of astroglial NF-kappaB enhances oligodendrogenesis following spinal cord injury
Source: J Neuroinflammation. 2013 Jul 23;10:92. doi: 10.1186/1742-2094-10-92 (PMC3751509; doi:10.1186/1742-2094-10-92)

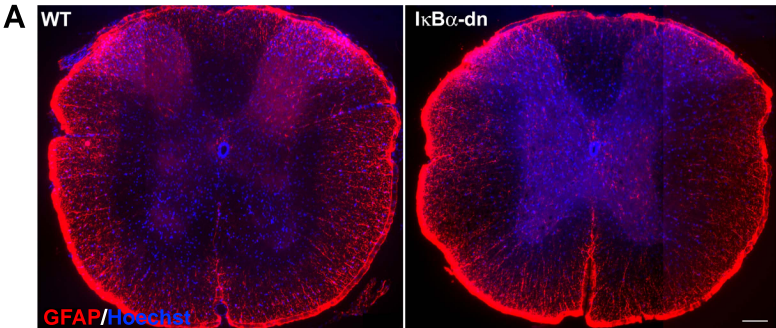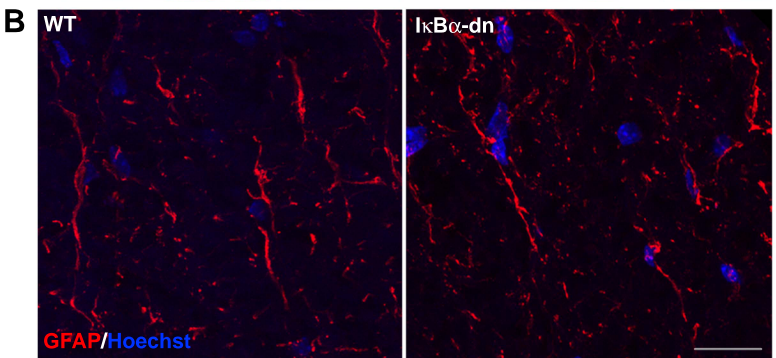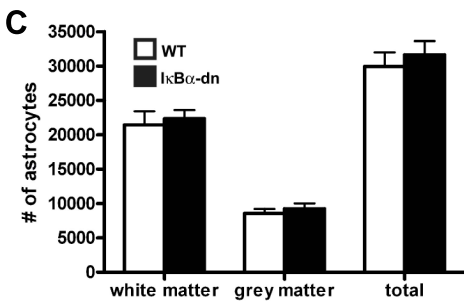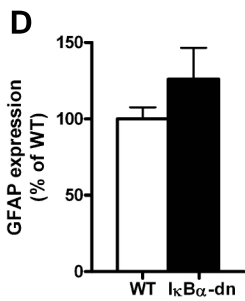

Supplement: Additional file 1 — Inhibition of astroglial NF-κB does not affect the number of astrocytes in the naïve, murine adult spinal cord. (A) Representative immunostained spinal cord cross sections from naïve wild-type (WT) and IκBα-dn transgenic (TG) mice. Astrocytes were immunostained using a polyclonal rabbit anti-GFAP (DAKO, 1:1000) and an Alexa594 anti-rabbit secondary antibody (Molecular Probe, 1:500). Hoechst was used to label the nuclei. Scale bar: 100 μm. (B) High magnification of astrocytes in the white matter spinal cord of WT and IκBα-dn TG mice. Scale bar: 20 μm. (C) Estimation of the number of astrocytes in the white matter and grey matter of a 1-mm long spinal cord segment in the thoracic region of naïve WT and IκBα-dn mice using unbiased stereology (grid size 120 μm × 120 μm and probe size 40 μm × 40 μm) showed no difference between genotypes (mean ± SEM, N = 3 per group). (D) Glial fibrillary acidic protein (GFAP) gene expression level in the spinal cord was assessed by real-time PCR. Data were normalized to β-actin and expressed as percent of WT (mean ± SEM, N = 5 per group). [file 1742-2094-10-92-S1.pdf]
